# Evidence for viable and stable triploid *Trypanosoma congolense* parasites

**DOI:** 10.1186/s13071-017-2406-z

**Published:** 2017-10-10

**Authors:** Eliane Tihon, Hideo Imamura, Jean-Claude Dujardin, Jan Van Den Abbeele

**Affiliations:** Department of Biomedical Sciences, Institute of Tropical Medicine, Nationalestraat 155, 2000 Antwerp, Belgium

**Keywords:** *Trypanosoma congolense*, Triploidy, Whole genome sequencing

## Abstract

**Background:**

Recent whole genome sequencing (WGS) analysis identified a viable triploid strain of *Trypanosoma congolense*. This triploid strain BANANCL2 was a clone of the field isolate BANAN/83/CRTRA/64 that was collected from cattle in Burkina Faso in 1983.

**Results:**

We demonstrated the viability and stability of triploidy throughout the complete life-cycle of the parasite by infecting tsetse flies with the triploid clone BANANCL2. Proboscis-positive tsetse flies efficiently transmitted the parasites to mice resulting in systemic infections. WGS of the parasites was performed at all life-cycle stages, and a method based on a block alternative allele frequency spectrum was developed to efficiently detect the ploidy profiles of samples with low read depth. This approach confirmed the triploid profile of parasites throughout their life-cycle in the tsetse fly and the mammalian host, demonstrating that triploidy is present at all stages and is stable over time.

**Conclusion:**

The presence of viable field-isolated triploid parasites indicates another possible layer of genetic diversity in natural *T. congolense* populations. The comparison between triploid and diploid parasites provides a unique model system to study the impact of chromosome copy number variations in African trypanosomes. In addition, the consequences of triploidy can be further investigated using this stable triploid model.

**Electronic supplementary material:**

The online version of this article (10.1186/s13071-017-2406-z) contains supplementary material, which is available to authorized users.

## Background

African trypanosome genomes are considered to be diploid [1, 2]. Yet, triploid *T. brucei* parasites have been observed following experimental crossing [3–5]. It has been postulated that triploid *T. brucei* parasites are atypical but not lethal [6], and that they resulted from either the fusion between diploid and haploid nuclei, or from failures during meiosis due to parental strains possessing very divergent homologous chromosomes [5, 7].

Meiosis in African trypanosomes is a complex process so far only described for *T. brucei*. Non-obligatory mating of *T. brucei* [5] occurs in the tsetse fly salivary glands where haploid gametes can be found and meiosis-specific genes are expressed [5, 8–10]. This is the life-cycle stage at which triploid *T. brucei* potentially emerge. However, no triploid strains of *T. brucei* have been reported from the field, suggesting that viable triploids are rare or have been frequently overlooked.

In a previous phylogenomic study of *T. congolense*, we discovered a triploid strain isolated from infected cattle in Burkina Faso which represented the first documented case of triploidy in a field-isolated African trypanosome [11]. There is currently no further information about its frequency in natural trypanosome populations or its persistence and impact on its viability throughout its life-cycle. Moreover, to understand the underlying mechanisms of triploidy emergence in *T. congolense*, further knowledge about the existence of a putative sexual stage during the parasite life-cycle is required.

In the *T. congolense* life-cycle, the presence of a sexual stage with the expression of meiosis-specific genes has currently not been formally identified. However, recent genetic studies on *T. congolense* field populations clearly indicated the existence of recombination between different parasite populations, suggesting the existence of a sexual stage in the species life-cycle [11].

In this study, we monitored the ploidy status of the *T. congolense* clone BANANCL2 during its development in the tsetse fly and the mammalian host following an infected tsetse bite. We found that triploidy is a stable trait that persists throughout the parasite life-cycle; the triploid parasites are viable at all stages and fully capable of infecting the murine host when they were inoculated through tsetse fly bite.

## Methods

### Origin of the samples and parasite isolation from mouse blood

The *T. congolense* savannah strain BANAN/83/CRTRA/64 was collected in 1983 in Burkina Faso [12]. The clones BANANCL1, BANANCL2 and BANANCL3 were obtained after infecting rodents with a single parasite of the BANAN/83/CRTRA/64 strain using the micro-drop method [13]. The savannah strain KTT/MSOROM7C1 (referred to as MSOROM7 in this study) was isolated in Zambia in 2003 [13]. The savannah strain MBOI/BK/89/SA268 (referred to as SA268 in this study) was isolated in Burkina Faso in 1989 [14]. MSOROM7 was selected because of its high depth coverage (i.e. over 84×) which was suitable for computational simulation for mixing diploidy and triploidy samples. SA268 was selected because it was genetically very similar to BANANCL1. All parasite strains were morphologically indistinguishable.

The *T. congolense* strains were maintained in OF-1 mice. The isolation of parasites from mouse blood was performed as described previously [11].

### Tsetse flies infections and parasite extraction

*Glossina morsitans morsitans* were obtained from a colony at the Institute of Tropical Medicine, Antwerp, Belgium. Their origin and rearing conditions are described elsewhere [15].

Tsetse fly blood meals were prepared using mouse blood infected with *T. congolense* BANANCL2. This was diluted to a final concentration of 2.5 × 10^6^ tryp/ml in 15 ml defibrinated horse blood and supplemented with 10 mM reduced L-glutathione. Freshly emerged tsetse flies were then fed 24 h after emergence. Afterwards, flies were maintained for 28 days by feeding them 3 times a week on clean defibrinated horse blood. At the end of the experiment, the midgut, proventriculus and proboscis of each fly was dissected and parasites were collected for DNA extractions. Three independent infection experiments were performed. Before dissection, some proboscis-infected tsetse flies were used to probe on anaesthetized mice, resulting in systemic blood infections [16]. Parasites from these infected mice were collected for DNA extraction as described previously [11].

### DNA extraction, high-throughput sequencing, read mapping and variant calling

DNA extractions were performed using the QIAamp DNA Blood Mini Kit (Qiagen, Hilden, Germany) according to manufacturer’s instructions. Whole genome sequencing (WGS) experiment and analyses were performed as mentioned previously [11].

### Estimating allele frequency profile from SNP blocks

In the WGS, the DNA strands are sequenced randomly and a high depth coverage will ensure that each DNA strand (and their corresponding nucleotide sequences) is well represented in the analysis. In this case the alternative allele frequency is calculated as the ratio of read depth of an alternative allele over the total read depth at heterozygous SNP sites. This is similar to the method described in [17]. However, the accurate estimation of the alternative allele frequency distribution of a sample with a read depth coverage lower than 30× is hampered because the proportion of each DNA strand may not be equally represented in the sequence analysis, resulting in an uneven SNP distribution for the SNP variants. This problem is more pronounced in a triploid sample because of the presence of an additional DNA strand. Normally, triploid samples with high read depth show two peaks at 0.33 and 0.66 in their alternative allele frequency distribution [17]. Due to larger uncertainty for allele frequency estimation for low read depth samples, these two peaks start to overlap, making it impossible to distinguish their ploidy status unambiguously.

Therefore, to overcome this problem we developed an alternative allele frequency calculation method based on blocks of 1000 SNP sites. Here, both heterozygous and homozygous SNP sites were included since they could not be properly distinguished at low depth sites. This method aggregates the read depth information of multiple read positions (i.e. 1000 SNP sites) for better allele frequency estimation. For all of the 1000 individual SNP sites of each block, we summed up separately the frequency of alleles corresponding to the reference bases (Ref) and the frequency of alleles corresponding to the alternative bases (Alt). The alternative allele frequency of each block was then estimated using the formula Alt / (Ref + Alt). This provided an aggregate alternative allele frequency for a given block (i.e. the block alternative allele frequency) that reflected the proportion of homozygous and heterozygous sites found within the block (Additional file 1: Figure S1). For instance, a block alternative allele frequency close to 1 suggests that most of the 1000 SNP sites within the block are homozygous. Likewise, a block alternative allele frequency of 0.8 suggests a combination of mostly homozygous SNP sites and few heterozygous SNP sites. Other frequency values could reflect a combination of multiple SNPs of different heterozygosity. For instance, in a triploid sample, a frequency of 0.5 could represent a block entirely heterozygous with 50% of the SNPs with a frequency of 0.33 and 50% of the SNPs with a frequency of 0.66; or could reflect a combination of heterozygous and homozygous SNP sites (Additional file 1: Figure S1). We combined the block alternative allele frequencies and obtained a profile of aggregate alternative allele frequencies for all the chromosomes.

We performed the block alternative allele frequency calculation for BANAN/83/CRTRA/64, BANANCL1, BANANCL2, BANANCL3, the 16 BANANCL2 samples from fly tissues and tsetse-infected mouse blood, and SA268. In our analysis, BANANCL2, which had the highest read depth and provided the most reliable allele frequency estimation, was set as standard triploid sample, and SA268 was set as standard diploid sample. It should be noted that these profiles are strain-specific and could only be used to determine the ploidy profiles of genetically similar strains. In addition, we calibrated the block alternative allele frequency method for various depths using different fractions of the BANANCL2 sample. We created the block alternative allele frequency profiles of a depth range of 79×, 44.5×, 22.3×, 13.4×, 10.4×, 5.3×, 1.15×, 0.58×, 0.27× to 0.1× (i.e. using 50–0.1% of the sample depth coverage). The resulting allele frequency profiles were consistent with those obtained with a sufficient depth coverage, indicating the accuracy of this technique to estimate the allele frequency profiles for samples with lower depth coverage. In addition, we saw that the profile of BANAN-PB-2 (i.e. lowest depth coverage of the dataset), corresponded to the profiles of BANANCL2 fractions with depths between 1.15 and 0.27 (data not shown).

### Flow cytometry

The DNA contents of BANANCL2 and MSOROM7 (i.e. diploid strain) were measured by flow cytometry using the propidium iodide flow cytometry kit (Abcam; λ_ex_: 493 nm; λ_em_: 636 nm) according to the manufacturer’s instructions. Briefly, parasites were DEAE-purified from OF-1 mice blood. For each condition, 10^7^ trypanosomes were fixed and permeabilized overnight with 70% ice-cold methanol. The parasites were washed twice with Phosphate Buffer Saline (PBS), and stained with a solution containing 50 μg/ml propidium iodide and 550 U/ml ribonuclease A. The parasites were incubated for 30 min at RT in the dark and were analyzed with a BD FACSVerse flow cytometer and the BD FACSuite software v1.0.3.

## Results

### Predominance of the triploid population in a mixed *T. congolense* infection

**Fig. 1:**
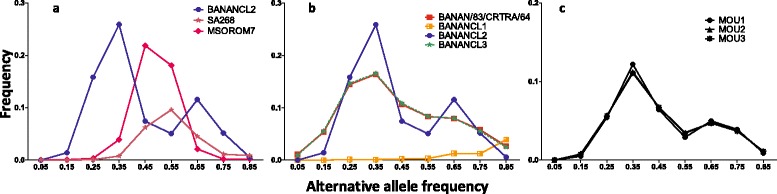
Alternative allele frequency profiles based on 11 chromosomes. The alternate allele frequencies are shown for: **a** the diploid strains SA268 and MSOROM7 and the triploid clone BANANCL2; **b** BANAN/83/CRTRA/64 parasites and its clones BANANCL1, BANANCL2 and BANANCL3; **c** the BANANCL2 parasites extracted from the blood of tsetse-bite infected mice after completion of the full parasite cycle in the tsetse vector. The distribution of read depths for alternative alleles at heterozygous sites showed two peaks at around 0.33 and 0.66 for triploid chromosomes, and one peak at around 0.5 for diploid chromosomes. Note, these alternative allele frequencies were calculated based on each heterozygous SNP site for all chromosomes

We previously discovered a triploid *T. congolense* strain that showed a hybrid profile between parasites from both Zambia and Burkina Faso [11]. This sample, BANANCL2, was a clone of the BANAN/83/CRTRA/64 isolate that was collected from cattle in 1983 in Burkina Faso [12]. The triploid status of the BANANCL2 clone was identified by measuring the alternative allele frequencies at heterozygous sites based on WGS data [11]*.* In a diploid strain, the alternative allele frequency distribution is bell shaped with its peak around 0.5 for each chromosome, as observed in the *T. congolense* diploid strains SA268 and MSOROM7 (Fig. 1a). In contrast, the allele frequency distribution of BANANCL2 is completely different with two distinct peaks at around 0.33 and 0.66 for each chromosome which is characteristic of triploidy (Fig. 1a) [17].

**Fig. 2:**
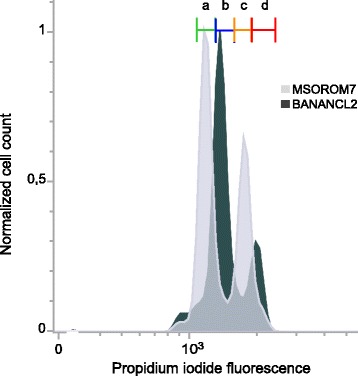
DNA content of MSOROM7 and BANANCL2 determined by flow cytometry using a propidium iodide staining. **a** 2 N cells. **b** 3 N cells. **c** 4 N cells. **d** > 4 N cells. Propidium iodide fluorescence in arbitrary units (X axis) against normalized cell count (Y axis)

We further confirmed the triploid status of the BANANCL2 clone by quantifying and comparing its DNA content to that of the diploid MSOROM7 strain by flow cytometry (Fig. 2). The DNA content was measured on fixed and permeabilized cells stained with propidium iodide as described in [5]. In the diploid MSOROM7, the first major peak (a) corresponded to 2 N DNA cells in the G1/G0 cell cycle stage, and the second peak (c) to cells with a 4 N DNA content (i.e. G2 stage prior to cell division). For the BANANCL2 clone, the major peak (b) corresponded to cells with a 3 N DNA-content (i.e. between the 2 N and 4 N peaks of MSOROM7 cells) whereas the second peak (d) reflected a > 4 N DNA-content. This result confirmed the triploid status of BANANCL2 that was observed by WGS analysis.

To assess whether the triploid status of BANANCL2 was a stable trait already present in the original field sample, or whether it was acquired during the cloning procedure, we sequenced the whole genome of the original BANAN/83/CRTRA/64 field isolate and its other derived clones BANANCL1 and BANANCL3, and estimated their alternative allele frequency profiles. The alternative allele frequency spectra of both BANAN/83/CRTRA/64 and BANANCL3 showed a clear peak at 0.33 and a second less-pronounced peak at 0.66, while the alternative allele frequency profile of BANANCL1 did not highlight specific profile due to its low depth coverage (Fig. 1b, Table 1).

**Table 1:** Sequencing data. Read depth information of BANANCL2 life-cycle stages mapped to the *T. congolense* IL3000 reference genome following WGS. The parasites were collected from the tsetse midgut (MG), proventriculus (PV), proboscis (PB) and from the infected mouse after the tsetse fly bite (MOU)

	Average depth	Total reads	Mapped reads	
		(M)	(M)	(%)
BANAN/83/CRTRA/64	19.2	2.61	2.51	96.0
BANAN-MG-1	3.5	2.21	0.39	17.7
BANAN-MG-2	7.4	3.35	0.86	25.5
BANAN-MG-3	3.9	3.65	0.47	12.9
BANAN-PB-1	1.9	3.62	0.23	6.4
BANAN-PB-2	1.7	3.39	0.13	3.9
BANAN-PB-3	2.7	3.07	0.30	9.9
BANAN-PV-1	3.3	3.04	0.41	13.6
BANAN-PV-2	2.6	2.76	0.26	9.6
BANAN-PV-3	19.2	3.79	2.13	56.1
BANAN-MOU-1	36.2	4.42	3.69	83.4
BANAN-MOU-2	31.5	3.86	3.21	83.2
BANAN-MOU-3	35.1	4.65	3.78	81.3
BANANCL1	8.9	1.70	1.60	93.7
BANANCL2	79.0	11.45	10.92	95.4
BANANCL3	24.6	4.23	3.98	94.0

*M* million

**Fig. 3:**
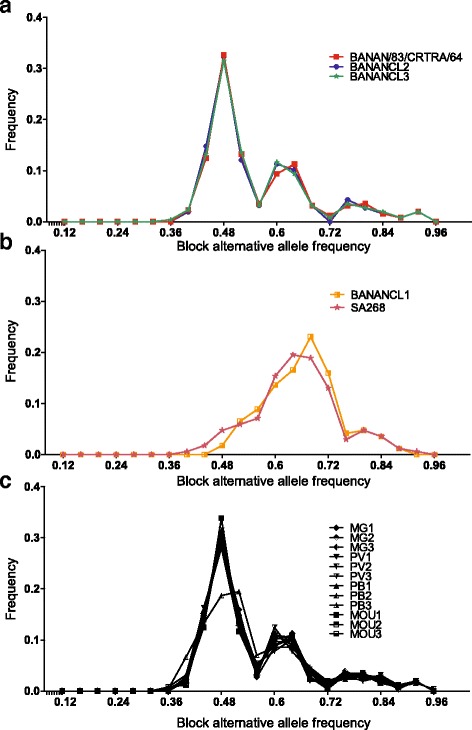
Block alternative allele frequency profiles. The block alternative allele frequency profiles based on windows of 1000 SNPs are shown for all the different BANAN samples. This technique enabled the estimation of the alternate allele frequencies in samples with low read depth. **a** The block frequency profile of BANAN/83/CRTRA/64, BANANCL3 and BANANCL2. **b** The block frequency profile of the diploid Burkinabe sample *T. congolense* SA268 is shown here as diploid control. **c** All the BANANCL2 samples showed a frequency profile characteristic of triploid samples (see main text and Methods). In contrast, BANANCL1 showed an alternate allele frequency profile with one peak characteristic to diploid samples. *Abbreviations*: MG, midgut; PV, proventriculus; PB, proboscis; MOU, mouse

For the calculation of allele frequencies from samples with average depth coverage lower than 30×, we estimated the alternative allele frequencies based on blocks of 1000 SNP sites instead of on individual heterozygous SNP sites. We then obtained an average alternative allele frequency for a given SNP block (see Methods). We first determined the profile of the block alternative allele frequencies of the clone BANANCL2 and the strain SA68 to establish the reference base line profile of a triploid and diploid parasite population, respectively (Fig. 3a, b; Additional file 1: Figure S1). Using this approach, we identified that BANAN/83/CRTRA/64 and BANANCL3 were triploid, as characterized by a block alternative allele frequency profile similar to BANANCL2 (Fig. 3a) (i.e. correlation values of *r* ^2^ = 0.996 and *r* ^2^ = 0.997, respectively; Table 2). In contrast, BANANCL1 had a block alternative allele frequency profile similar to SA268 and was therefore classified as diploid (Fig. 3b). The identification of one diploid clone indicated that the original BANAN/83/CRTRA/64 parasite population was in fact a mixed infection between triploid and diploid parasites. We tried to estimate the proportion of BANANCL1 in BANAN/83/CRTRA/64 based on specific homozygous SNP markers. However, at each individual SNP level, BANAN/83/CRTRA/64 and BANANCL2 were very similar and there were no BANANCL1 specific homozygous SNP markers that separated BANANCL1 from BANAN/83/CRTRA/64 and BANANCL2. Therefore, the proportion of BANANCL1 in the BANAN/83/CRTRA/64 sample was undetectably low, suggesting that BANANCL1 was not a dominant population in the BANAN/83/CRTRA/64 isolate. In addition, the similarity between the block alternative allele frequency profiles of BANAN/83/CRTRA/64 and BANANCL2 (i.e. correlation value of *r* ^2^ = 0.996) also indicated that the triploid population was the predominant population found in this isolate.

**Table 2:** Correlation values between the block alternative allele frequencies of the parasites in the three mouse groups and their respective parasite stages in the different tsetse fly tissues

ID	ID	*r*	*r* ^2a^	*P-*value^b^
BANAN/83/CRTRA/64	BANANCL2	0.998	0.996	< 0.0001
BANANCL1	BANANCL2	0.585	0.342	< 0.0001
BANANCL3	BANANCL2	0.998	0.997	< 0.0001
BANAN-MOU-1	BANAN-MG-1	0.994	0.988	< 0.0001
BANAN-MOU-1	BANAN-PB-1	0.988	0.975	< 0.0001
BANAN-MOU-1	BANAN-PV-1	0.991	0.981	< 0.0001
BANAN-MOU-2	BANAN-MG-2	0.996	0.992	< 0.0001
BANAN-MOU-2	BANAN-PB-2	0.969	0.938	< 0.0001
BANAN-MOU-2	BANAN-PV-2	0.989	0.978	< 0.0001
BANAN-MOU-3	BANAN-MG-3	0.992	0.983	< 0.0001
BANAN-MOU-3	BANAN-PB-3	0.991	0.982	< 0.0001
BANAN-MOU-3	BANAN-PV-3	0.997	0.995	< 0.0001

^a^The coefficients of determination *r* ^2^ are high between BANANCL2, BANAN/83/CRTRA/64 and BANANCL3, as well as between the tsetse-bite infecting mice samples and the tsetse samples, indicating that the overall allele frequencies remained triploid in all these groups. In contrast, the coefficient of determination *r* ^2^ is low between BANANCL1 and BANANCL2, indicative of their genomic differences

^b^The *P*-values were calculated by a two-tailed test

*Abbreviations: MG* midgut, *PV* proventriculus, *PB* proboscis, *MOU* mouse

### Maintenance of triploidy throughout the *T. congolense* life-cycle

We next determined the ploidy status of the BANANCL2 strain throughout its life-cycle after infections of *G. morsitans morsitans* tsetse flies (i.e. three groups of independently infected flies). At 28 days post-infection, proboscis-positive tsetse flies were detected in all three groups. The bite of these infected flies successfully resulted in a systemic infection of mice. Parasites were isolated for WGS analyses from the midgut, proventriculus and proboscis of the flies, and from the blood of infected mice. These isolates represented the main life-cycle stages of *T. congolense*. Paired-end reads were mapped against the reference *T. congolense* IL3000 genome [18]. The average depth coverage was 4.9×, 8.4× and 2.1× for parasites extracted from the tsetse fly midgut, proventriculus and proboscis, respectively (Table 1). The average depth coverage was 34.3× for parasites isolated from the murine hosts. The depth coverage of parasites isolated from flies was low, and on average only 17.3% of the reads could be mapped to the *T. congolense* reference genome because of the difficulty in extracting pure parasite fractions without any tsetse tissue contaminations.

Despite the lower read depth for these samples, we were able to estimate the ploidy of parasite populations isolated from the different tsetse fly tissues, and from the blood of the infected mice, using the block alternative allele frequency method. We observed that the alternative allele frequency profiles were all similar to the frequency profile of the triploid sample BANANCL2 (Fig. 3c), except for one proboscis-derived parasite (BANAN-PB-2) which had a very low depth coverage (i.e. 1.7× coverage; Table 1). Nevertheless, we identified that this profile corresponded to a triploid sample with lower depth by using additional verifications (see Methods). The read depths of the blood stream parasites extracted from the infected mice were > 30× (Table 1). We were therefore able to identify their triploid patterns from individual chromosomes using the alternative allele frequency spectra based on individual SNP sites and observed two distinct peaks around 0.33 and 0.66 (Fig. 1c).

To provide statistical significance of the outcome of the block alternative allele frequency method for the low read depth samples, we calculated the coefficient of determination (*r* ^2^) between the block alternative allele frequencies of the parasites in the three mice groups (i.e. we confirmed triploidy based on the alternative allele frequency profiles, Fig. 1c) and their respective parasite stages in the different tsetse fly tissues. All *r* ^2^ values were found to be high, indicating that the overall allele frequencies remained triploid in all parasite stages (Table 2). In contrast, the *r* ^2^ values between the block alternative allele frequencies of the diploid strain BANANCL1 and the triploid strain BANANCL2 was low, reflecting the genetic differences between both strains (Table 2).

Finally, we analyzed the genome sequence data from the different BANANCL2 parasite life-cycle stages in order to look for genetic signatures of recombination in the parasites, such as long losses of heterozygous sites or long traces of shifts in SNP heterozygosity, as previously observed in WGS analyses [11, 19]. The presence of such genetic signatures at a specific *T. congolense* life-cycle stage would be indicative of an elevated recombination activity at that stage and could in turn reflect a potential site of parasite sexual exchange where triploid cells can emerge accidentally. However, we found no evidence of large genomic changes and did not observe genomic footprints of genetic recombination.

## Discussion

The genome of African trypanosomes is reported to be diploid [1, 2]. However, our results demonstrated that triploid *T. congolense* parasites can complete a full life-cycle, and that triploid populations in the field are viable and sustained in host populations. We used a WGS approach to estimate the state of ploidy of *T. congolense* populations during each stage of the parasite life-cycle. We then developed a method based on the block alternative allele frequency to accurately detect the ploidy profiles of samples with low sequencing depth. This method could be extended to other organisms and low parasitemia samples including those directly collected in the field for ploidy estimation.

When analyzing the ploidy status of the field isolate BANAN/83/CRTRA/64 and its clones BANANCL1, BANANCL2 and BANANCL3, we found that BANAN/83/CRTRA/64 was a mixed infection between diploid and triploid populations, with the triploid population predominating. From this, we hypothesize that the triploidy that emerged in this BANAN natural population conferred a beneficial advantage to the diploid counterpart in their local ecological/epidemiological setting.

The advantages and disadvantages of polyploidy have been reported in various organisms. For instance, advantages associated with changes in ploidy have been described in *Leishmania* parasites [20, 21], in *T. cruzi* parasites [22, 23], in yeasts [24], and in the fungal pathogen *Cryptococcus neoformans* [25, 26]. In *Leishmania major*, changes in the number of particular chromosomes in the genome (i.e. aneuploidy) resulted in gene amplifications and were associated with drug resistance [20, 21]. In yeast, whole-genome duplication (i.e. polyploidy) has been shown to play a key role in their evolutionary adaptation to stressful conditions [24]. Polyploidy increased the genetic diversity of the yeast population contributing to the acquisition and spread of beneficial mutations. Similarly in *C. neoformans*, rates of survival and reproduction of polyploid cells were better under stressful conditions in comparison to their haploid counterparts [25].

The increase in ploidy enlarged the size of the genome, enhancing the content and diversity of the gene pool. Some of the newly acquired genes might confer phenotypic advantages to the strain, such as resistance to a drug, adaptation to stressful conditions, or changes in virulence and pathogenicity.

Triploidy could also be associated with reduced viability and reduced chances for a strain to complete a full life-cycle because of replication costs associated with the increased DNA size of the genome [5]. However, this does not seem to be the case here since the triploid population in the original field isolate surpassed the diploid population, suggesting that its increased DNA size did not affect its capability to establish a systemic infection.

Since we have isolated one triploid sample out of 56 *T. congolense* parasites sequenced [11], we suspect that triploidy may play a larger role in *T. congolense* natural populations as currently assumed. The emergence of triploid and other polyploid trypanosome cells during experimental crossings between *T. brucei* parasites is not uncommon [5], and it can be assumed that a similar scenario is also happening in *T. congolense*. However, whether our observation of triploidy in a natural *T. congolense* population is an isolated case or occurs more frequently remains to be verified.

To date, the frequency of triploid *T. congolense* parasites in natural populations is unknown partly because few studies have been designed for its identification. For instance, identifying triploidy with routine approaches, such as microsatellites genotyping, is not practical since a triploid profile is nearly identical to profiles of mixed infections. However, the emergence of Next Generation Sequencing technologies will greatly improve the detection of these parasite populations in future, and will provide further information about their frequency.

Triploid cells could emerge due to errors during meiosis. We previously demonstrated the presence of recombination breakpoints in the genome of the original BANANCL2 line [11]. Here, we analyzed whole genome sequence data extracted from BANANCL2 at each life-cycle stage to identify recombination signatures and determine the life-cycle stage at which recombination, and therefore a potential emergence of triploidy, is taking place in *T. congolense*. However, there was no genomic evidences for new recombination events in our collection of samples, partly because recombination between clonal cells is difficult to detect. It would be informative to further explore meiosis in *T. congolense* and perform experimental crossings as described in *T. brucei* [5] to further address the potential link between meiosis and triploidy.

In addition to failures during meiosis, the triploid parasite population could also have emerged due to errors during mitosis (reviewed in: [27]). Here, errors at the different checkpoints of mitotic division could have led to improper chromosome segregation and the emergence of daughter cells with improper number of chromosomes.

## Conclusion

Our finding is a recent addition to increasing observations of changes in ploidy found in trypanosomatid parasites such as clinical and laboratory lines of *Leishmania* spp. [17, 21, 28, 29] and *T. cruzi* parasites [22, 23], highlighting its prevalence and significance in kinetoplastids. The triploid parasites identified in this study represent an alternative model system to study different aspects of the *T. congolense* biology, such as gene expression levels related to recombination, drug resistance and other stress responses. In addition, comparing triploid to diploid parasites can provide unique insights into the impact of chromosome copy number variations in *T. congolense* natural populations.

## Additional file

Additional file 1: Figure S1. Block alternative allele frequency method. We calculated the block alternative allele frequencies of the triploid strain BANANCL2 and of the diploid strain SA268. **a** Allele frequency of each individual SNP site for the BANANCL2 strain for all the chromosomes. We can observe that most heterozygous SNPs have a frequency of about 0.33 or 0.66. **b** Allele frequencies of blocks of 1000 SNP positions for the BANANCL2 strain for all the chromosomes. Here, we summed up the read depth information of blocks of 1000 SNP positions, including both heterozygous and homozygous sites, which resulted in an aggregate alternative allele frequency for a given block. **c** Block alternative allele frequency spectrum of BANANCL2 (see Fig. 3a). **d.** Allele frequency of each individual SNP site for the SA268 strain for all the chromosomes. We can observe that most heterozygous SNPs have a frequency of about 0.5. **e** Allele frequencies of blocks of 1000 SNP positions for the SA268 strain for all the chromosomes. **f** Block alternative allele frequency spectrum of SA268 (see Fig. 3b) (PDF 6921 kb)

## Abbreviations

MG: Midgut
MOU: Mouse
PB: Proboscis
PV: Proventriculus
WGS: Whole Genome Sequencing
