# Supplementary figures and images for: Evidence for viable and stable triploid Trypanosoma congolense parasites
Source: Parasit Vectors. 2017 Oct 10;10:468. doi: 10.1186/s13071-017-2406-z (PMC5635536; doi:10.1186/s13071-017-2406-z)

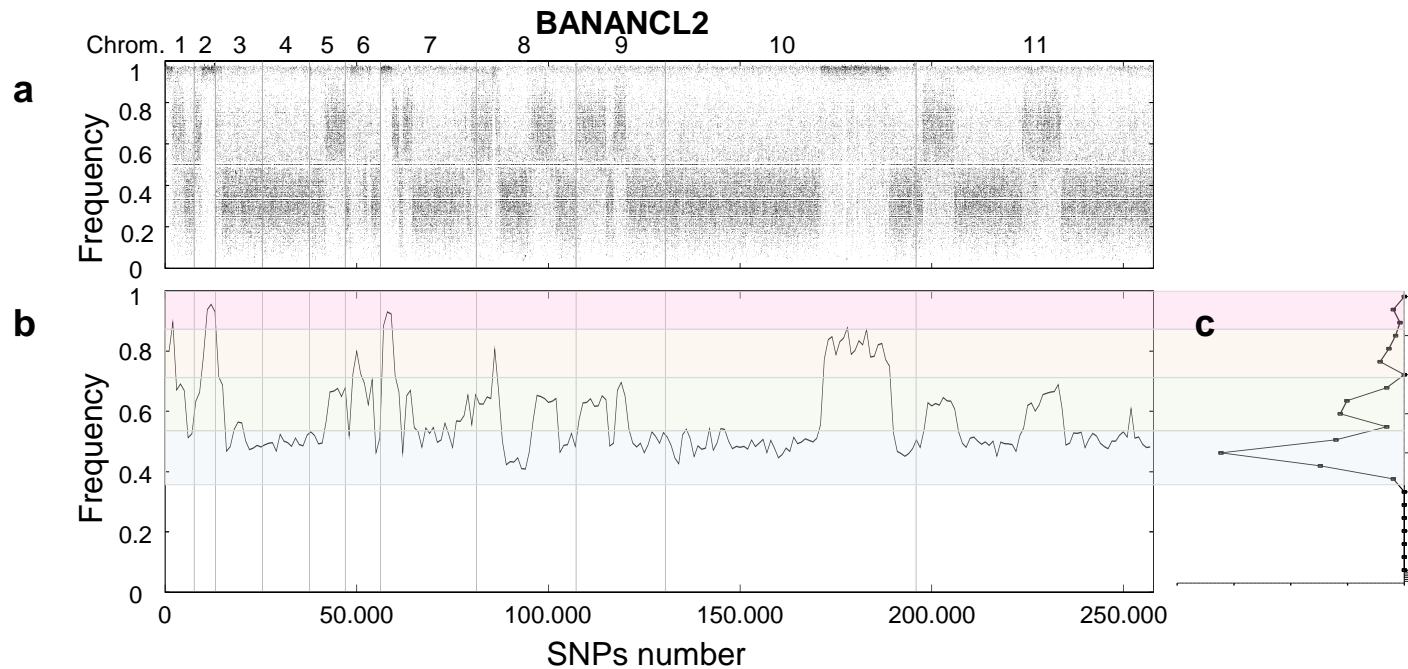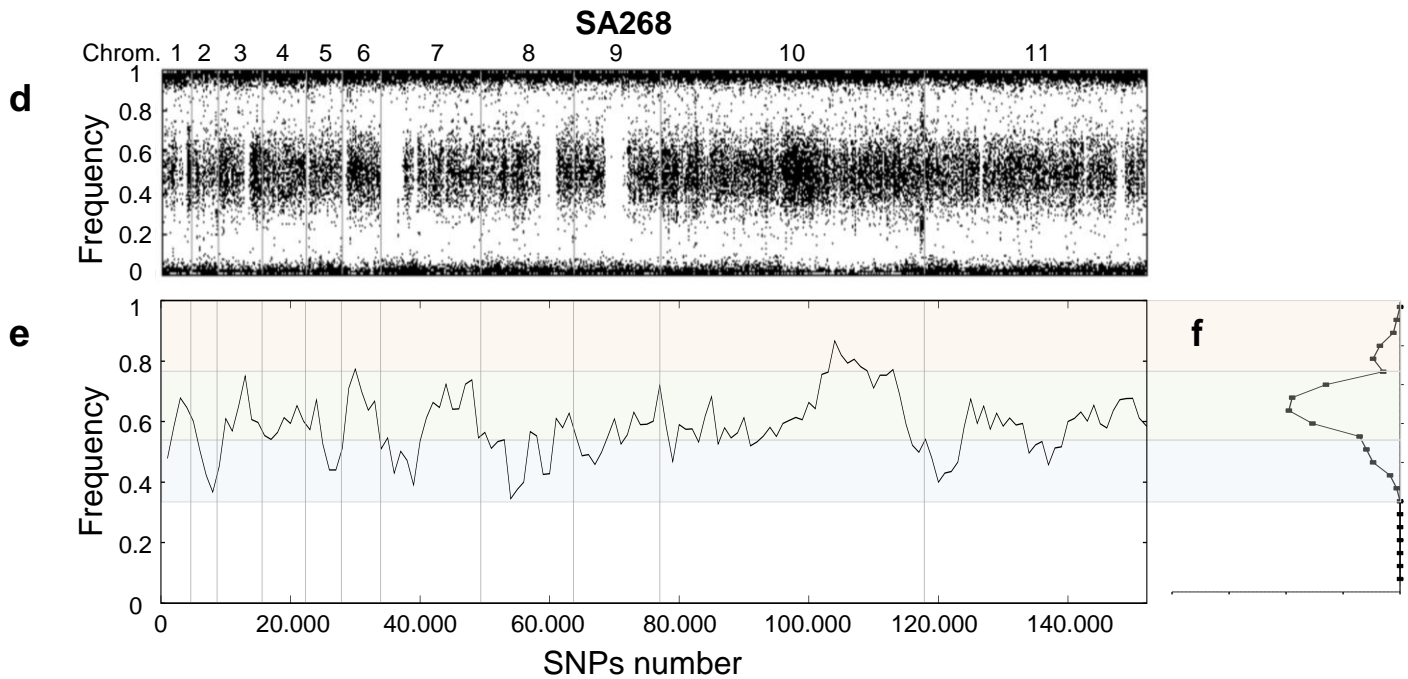

Supplement: Additional file 1: Figure S1. — Block alternative allele frequency method. We calculated the block alternative allele frequencies of the triploid strain BANANCL2 and of the diploid strain SA268. a Allele frequency of each individual SNP site for the BANANCL2 strain for all the chromosomes. We can observe that most heterozygous SNPs have a frequency of about 0.33 or 0.66. b Allele frequencies of blocks of 1000 SNP positions for the BANANCL2 strain for all the chromosomes. Here, we summed up the read depth information of blocks of 1000 SNP positions, including both heterozygous and homozygous sites, which resulted in an aggregate alternative allele frequency for a given block. c Block alternative allele frequency spectrum of BANANCL2 (see Fig. 3a). d. Allele frequency of each individual SNP site for the SA268 strain for all the chromosomes. We can observe that most heterozygous SNPs have a frequency of about 0.5. e Allele frequencies of blocks of 1000 SNP positions for the SA268 strain for all the chromosomes. f Block alternative allele frequency spectrum of SA268 (see Fig. 3b) (PDF 6921 kb) [file 13071_2017_2406_MOESM1_ESM.pdf]
